# Fast and Sensitive Determination of Iodide Based on Ternary Chalcogenides Nanoparticles

**DOI:** 10.3390/molecules29194751

**Published:** 2024-10-08

**Authors:** Zhitai Wang, Nengtao Wu, Weihao Wang, Yaozheng Hu, Zhijie Luo, Yuhui Zheng, Qianming Wang

**Affiliations:** 1 School of Materials Science and Engineering, Nanchang Hangkong University, Nanchang 330063, China; wangzt@nchu.edu.cn (Z.W.);; 2 School of Chemistry, Guangzhou Key Laboratory of Analytical Chemistry for Biomedicine, South China Normal University, Guangzhou 510006, China

**Keywords:** AgFeS_2_, nanocrystals, luminescence, fluorescence sensor, iodide ions

## Abstract

A fluorescent probe based on ternary AgFeS_2_ quantum dots has been prepared for the design of ternary chalcogenides. The nanoparticles are synthesized with oleylamine as a stabilizer at a low temperature (particle size in the range of 2 to 3 nm) and they exhibit an intense blue emission in aqueous media. As for their internal structure, each nanoparticle’s relative stoichiometric ratio (AgFe_1.01_S_1.91_) is very close to the theoretical value of 1:1:2. Their magnetic properties have been studied with a vibrating sample magnetometer and they have ferromagnetism between 4 K and 298 K (applied magnetic field ranging between −10,000 and 10,000 Oe). In the presence of iodide ions, the emission at 458 nm derived from AgFeS_2_ QDs has been observed to give rise to fluorescence quenching. The detection system is based on a static quenching process and morphological change between iodide ions and AgFeS_2_, which has a good linear range from 0 to 37.5 μmol/L, with a limit of detection of 0.99 μM. The nanoprobe responds within 30 s for the efficient detection of iodide. Such functional quantum dots will provide a powerful indicator in environmental and bio-sensing applications.

## 1. Introduction

Iodine is closely related to human growth and health as it is one of the essential trace elements in living life, playing pivotal roles in several biological processes, such as nutrition, neurological activity and thyroid gland function [1,2]. According to the World Health Organization, the daily intake of iodine is 150 μg or 250 μg in adults and during breast-feeding or pregnancy [3]. Both iodine (I^−^) deficiency and excessive intake can cause serious health problems, such as thyroid disease [4]. The insufficient supply of I^−^ may lead to fetal brain injury and increased infant mortality during pregnancy [5]. Therefore, the essential trace element of iodine is very important for the prevention of health issues. In many aspects of real life, the level of iodine in milk, salt and urine needs to be monitored to maintain the metabolism of thyroid diseases and the health of people. The determination of halide ion content has become the focus of our research. In previous studies, only a few papers reported the determination of iodide content through methods such as gas chromatography with mass spectrometry detection [6] and indirect atomic absorption spectrometry [7]. These methods are complicated and cumbersome. Therefore, it is of great significance to design and develop a fluorescence sensor system capable of rapid detection of iodide ions with high sensitivity, selectivity and convenience, as well as a low cost [8,9,10].

Due to its large ionic radius, low charge density and low hydrogen bonding capacity, iodide is the most difficult halide to detect in aqueous media; a recent review summarized the analytical techniques for the recognition of iodide [11]. Currently, diverse approaches have been employed for the determination of iodide, such as atomic absorption [12], gas chromatography [13], electrochemical analysis [14] and capillary electrophoresis [15]. Most of the current reported methods require sophisticated pre-treatment, complicated procedures, long operation times and costly equipment; therefore, the search for easy and low-cost detection methods is still challenging.

To date, numerous fluorescent sensors for the selective identification of iodide ions have been mainly created using the heavy atom effects and charge transfer of iodide. Previously, some small molecules of fluorescent sensors for detecting iodide ions were designed using organic synthesis [16,17] and carbon dots [18]. In recent years, a variety of probes including MOFs, COFs and alloys have been recorded [19,20,21,22,23,24,25,26,27].

It is well documented that the optical stability of the available organic chromophores in MOFs or COFs requires optimization, so the employment of functional materials derived from inorganic structures would be expected. With the development of nanotechnology, inorganic nanoparticle probes for ion detection have been developed [28,29]. Some ternary semiconductor materials are widely used for fluorescent probes, such as CsPbCl_3_, CuInS_2_ and AgInS_2_ quantum dots [30,31,32]. Compared with organic probes, inorganic nano fluorescent probes have a more stable intrinsic structure. In addition, frequently used semiconductor quantum dots such as CdS and PbS contain cadmium and lead. The element leaching problem would inhibit the application due to toxicity effects. Different from the traditional quantum dots, ternary chalcopyrites are totally free from Cd^2+^ or Pb^2+^ ions and these nanodomains could be considered as eco-friendly replacements. Moreover, inorganic nanoparticles are rarely used to detect iodide ions. Therefore, it is of great significance to design such nanoprobes for detection purposes. Fluorescence quenching would be caused by the combination of iodide ions with certain metal ions, which aggregate during recognition processes.

**Scheme 1:**
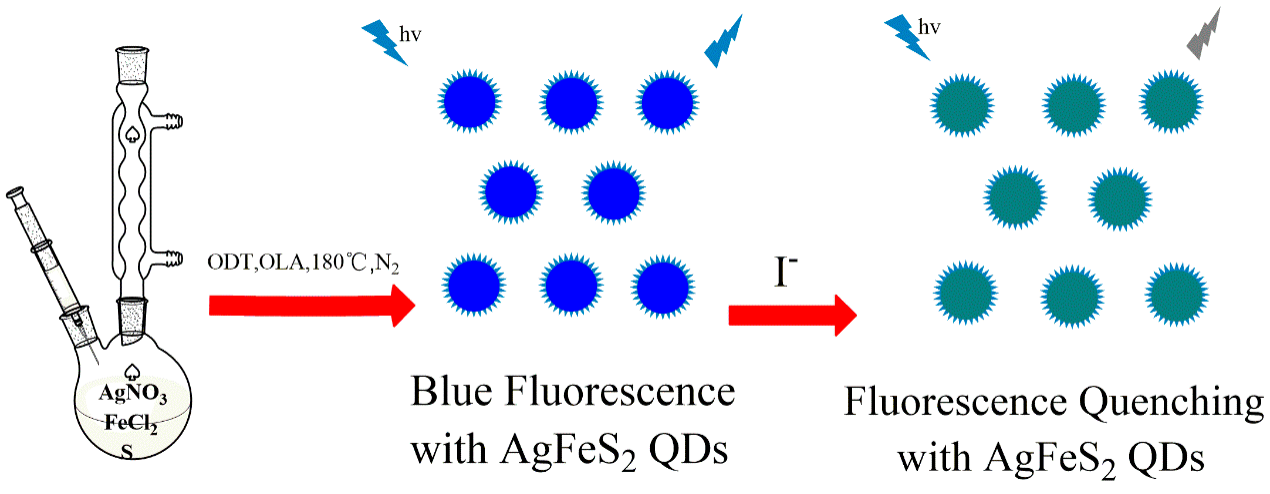
Schematic illustrations of the growth of AgFeS_2_ QDs via the one-step hot injection strategy and its application in I^−^ sensing (ODT: octadecanethiol; OLA: oleylamine).

The synthesis of a silver iron sulfide sensor for the selective and sensitive detection of I^–^ in aqueous solution has been provided in Scheme 1. The nanoparticle is synthesized with oleylamine as a stabilizer at a low temperature and exhibits an intense blue emission at 458 nm in aqueous media (ethanol:water = 1:4). Compared to previous studies, the method is simple and convenient as well as low-cost; in addition, no high temperatures or high pressure will be required. Further surface passivation is avoided. The facile strategy allows the possibility of scaled-up synthesis in the future. Remarkably, the AgFeS_2_ quantum dots have a narrow size distribution of around 2–3 nm and show good fluorescent stability in a wide range of acid and alkali conditions.

## 2. Experimental Section

### 2.1. Materials

Iron chloridetetrahydrate (FeCl_2_, 99.7%), silver nitrate (AgNO_3_, 99.8%), sulfur (S, 99%), oleylamine (C18:80–90%), 1-Dodecanethiol (98%), ascorbic acid (C_6_H_8_O_6_, 99.99%) and ethanol were purchased from Aladdin Chemistry Co., Ltd. (Shanghai, China). All the other reagents were purchased from Guangzhou Chemical Reagent Factory (Guangzhou city, China) and used without further purification.

### 2.2. Characterization

Photoluminescence spectra were measured by using a Hitachi-4600 spectrophotometer with a xenon lamp as the light source (Hitachi, Ltd., Tokyo, Japan). The scan speed was fixed at 1500 nm/min. Both excitation and emission slit widths were 5.0 nm. UV–visible spectra were collected by a Shimadzu UV-2700 spectrophotometer (Kyoto, Japan). X-ray diffraction (XRD) patterns were collected with a Japan Science Ultima IV X-ray Powder Diffractometer. TEM images were obtained with a JEOL JEM-2100HR transmission electron microscope. Particle size was also obtained with dynamic light scattering (DLS) using a particle size analyzer (Malvern Instrument, Malvern, UK). The magnetic properties of the AgFeS_2_ QDs were measured at room temperature using a vibrating sample magnetometer (VSM, BHV-50HTI). Lifetimes were measured with a Transient/Stationary Fluorescence Spectrometer (FLS-920, Edinburgh, UK). Element mapping images were shown through a Phenom Pro X Desktop Scanning Electron Microscope (Waltham, MA, USA). The full survey of XPS data was collected by an X-ray photoelectron spectrometer (Amicus, Shimadzu, Kyoto, Japan).

### 2.3. Synthesis of AgFeS_2_ Quantum Dots

FeCl_2_·4H_2_O (19.88 mg) and AgNO_3_ (16.99 mg) were added to a two-necked flask containing 1.5 mL oleylamine as a solvent. The reaction mixture was heated to 100 °C in a vacuum for 20 min to remove water. The mixture was then heated to 180 °C at a rate of 10 °C/min under nitrogen and was stirred for about 30 min. Then 1.5 mL of octadecanethiol (ODT) was added to the reaction mixture and the reaction mixture was heated at 180 °C to initiate the nucleation of AgFeS_2_ QDs. After adding the dodecanethiol, the solution color changed to bright yellow within a few seconds. The above solution was termed as sample-1.

Sulfur powder (40 mg) was added to a 50 mL three-necked flask containing 1.5 mL oleylamine. This flask was held at 160 °C in nitrogen for 30 min. Then, the sulfur in oleylamine was injected dropwise into the previous solution, sample-1 (representing the solution prepared in the above paragraph during the pre-treatment) and the mixture rapidly became black after the addition. Finally, the powder was collected by centrifugation, ultrasonically washed with deionized water and ethanol three times and air-dried at 70 °C for 24 h. As for the measurement of the pH values, the way to determine pH in mixed aqueous solvents (ethanol:water = 1:4) depends on differential potentiometry involving the employment of glass electrodes to detect protons and the reference electrode’s immersion in the solution. The pH values were obtained through the measured potential difference. The experiments were carried out at 25 °C with electrodes of the appropriate pH values after three-point calibration with typical aqueous buffer solutions (nominal pH, 4.005, 7.000 and 9.180).

## 3. Results and Discussion

**Figure 1:**
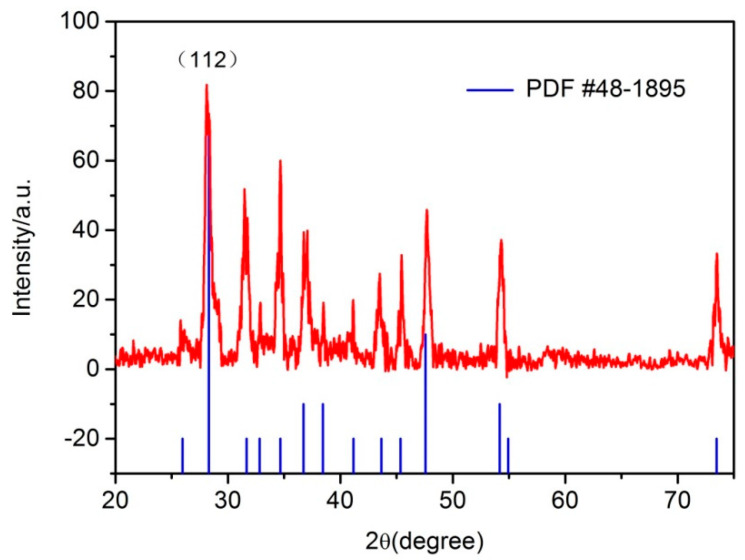
The X-ray diffraction (XRD) patterns of the obtained samples; the blue vertical is the standard literature data.

A simple one-pot route has been developed for the controlled synthesis of ternary AgFeS_2_ nanocrystals as precursors in oleylamine (OLA) at 180 °C. The X-ray diffraction (XRD) pattern of the AgFeS_2_ nanocrystals is shown in Figure 1, indicating that the sample can be indexed as a crystalline phase of AgFeS_2_ (JCPDS No. 65-2736). According to the curves, we use the following Debye–Scherrer formula to calculate the particle size [33].

$$D=\frac{K\gamma }{Bcos\theta }$$

**Figure 2:**
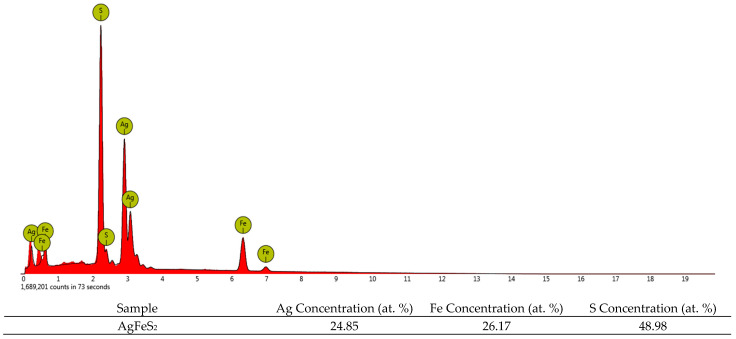
The EDX spectrum of AgFeS_2_ QDs (the bottom table represents the elemental composition of AgFeS_2_).

The range of this formula is 1–100 nm, so it is especially suitable for the crystal size calculation of nanomaterials. After calculation, the average crystallite size is about 5.24 nm. In the EDX spectrum analysis in Figure 2, the AgFeS_2_ quantum dots are composed of Ag, Fe and S elements. Elemental mapping (Figure S1) of the region containing the particles has been performed and the presence of three elements was confirmed. Quantitative determination of the data has been carried out and their contents were found to be 25.53%, 25.81% and 48.66% (Ag, Fe and S). The relative stoichiometric ratio has been measured as AgFe_1.01_S_1.91_, which is very close to the theoretical value of 1:1:2. The constitution and the oxidation state of AgFeS₂ have been explored (Figure S2). The full range analysis indicated the presence of Ag, Fe and S elements. The high resolution of the silver 3d spectra included the two signals of Ag 3d_5/2_ and 3d_3/2_, demonstrating that silver bears a +1 oxidation state. The binding energy levels of the Fe 2p_3/2_ and 2p_1/2_ peaks verified the +3 oxidation state. In the curves of sulfur, two bands of S 2p_1/2_ and 2p_3/2_ supported the presence of a −2 valence state for the sulfur element. Based on the results, the structural and valence information have been clarified.

**Figure 3:**
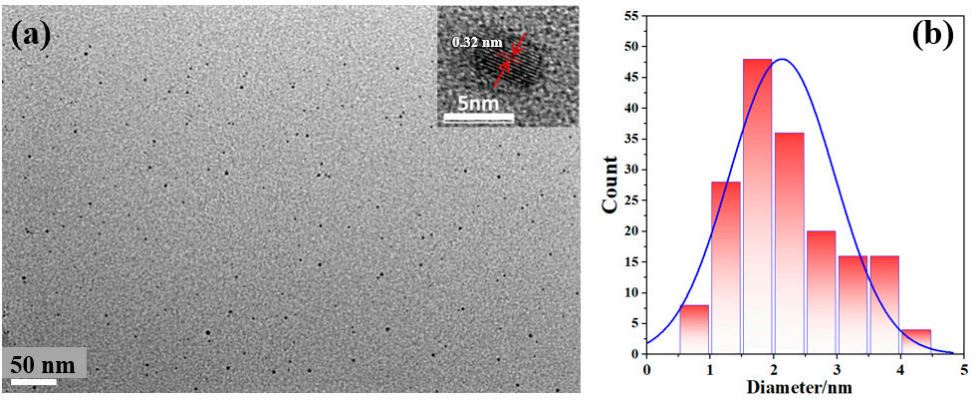
(**a**) HR-TEM images of the AgFeS_2_ QDs and (**b**) the DLS curve of the AgFeS_2_ QDs. The inset in the top right corner is the corresponding high-resolution TEM.

More information on the morphologies and crystals of the AgFeS_2_ QDs can be derived from transmission electron microscopy (TEM), as shown in Figure 3a. We can observe that these particles have better size dispersion and uniform distribution and the size of the AgFeS_2_ quantum dots was measured at approximately 2.65 nm. More importantly, the hydrodynamic size of the AgFeS_2_ QDs was measured using a particle size analyzer and the result, shown in Figure 3b, is consistent with TEM in the range between 2 and 3 nm. As expected, they display negative zeta potential (22.52 mV) due to the presence of –NH_2_ on the surface (Figure S3). Simultaneously, more crystal structure information has been obtained in the detailed inset image. Interplanar spacings of 0.32 nm are observed, which are well matched with the (112) lattice fringe of AgFeS_2_. This diffraction signal has also been observed in XRD analysis [34], so the microstructure studies of TEM images and the crystalline phase results from XRD are basically consistent.

**Figure 4:**
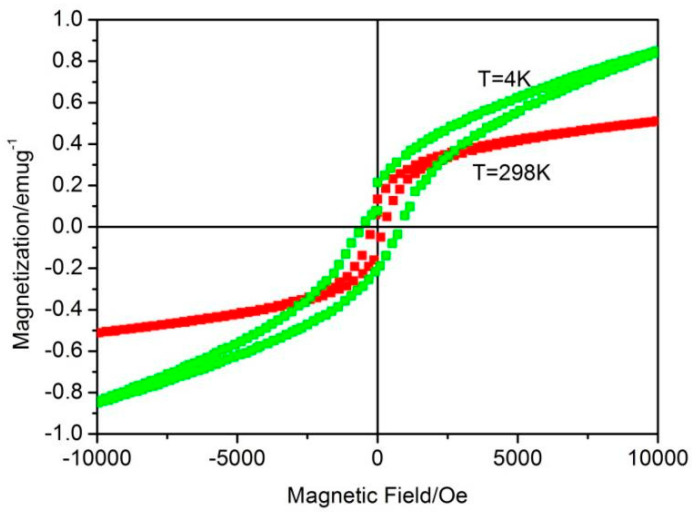
Magnetization curves of AgFeS_2_ QDs at 4 K and 298 K.

Vibrating sample magnetometry (VSM) is usually used to measure magnetic nanoparticles’ net magnetization [35] and it can measure the whole magnetization instead of that of a specific element [36]. To study the magnetic properties of the nanoparticles, the obtained hysteresis loops at 4 K and 298 K were measured between −10 k Oe and +10 k Oe and the content is provided in Figure 4. The ferromagnetic characteristics, including a magnetization value of 0.52 emu/g at 298 K and a magnetization value of 0.85 emu/g at 4 K, can be observed from Figure 4. From 298 K to 4 K during this process, the ferromagnetic features of the as-prepared sample were demonstrated. The results support the idea that this nanoplatform will provide an alternative approach to future utilization as an electromagnetic material or a contrast agent for MRI.

**Figure 5:**
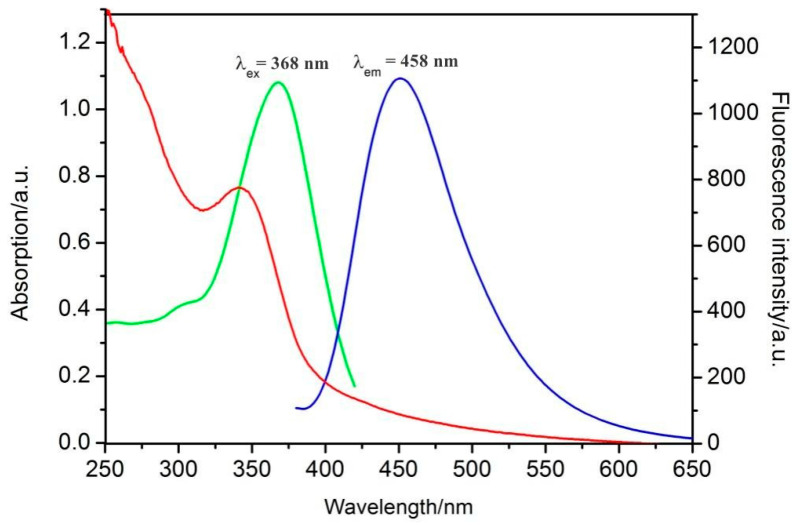
UV–visible absorption spectra of AgFeS_2_ QDs (red), PL excitation spectra (green) and PL emission spectra (blue) of the AgFeS_2_ QDs dispersed in ethanol:water = 1:4.

UV–visible absorption spectroscopy was used to study the optical properties of the AgFeS_2_ QDs. The absorption spectrum (Figure 5, red line) has been recorded and a peak at 340 nm is observed in the aqueous solution, which would be ascribed to the surface plasmon resonance signal derived from the formed silver species [37]. In Figure 5, the excitation signal has been measured and a very strong fluorescent blue emission (Figure 5, green line) was obtained at 458 nm, which was excited at 368 nm (Figure 5, blue line). As shown in Figure S4, the fluorescence intensity of the AgFeS_2_ QDs in aqueous solution exhibits a maximum fluorescence peak during excitation, evolving from 300 nm to 420 nm. Such a fact might be very important for further analysis of fluorescence sensing. Figure S5 shows the environmental effect on the fluorescence emission; the results reveal that the highest value of the AgFeS_2_ QDs’ fluorescence intensity was obtained at pH 5.0 in the range of 3.0–9.0. Therefore, pH 5.0 was selected as the condition in the next experiment. The pH stability in aqueous solution has been assessed and the emission can be maintained within a wide pH range.

**Figure 6:**
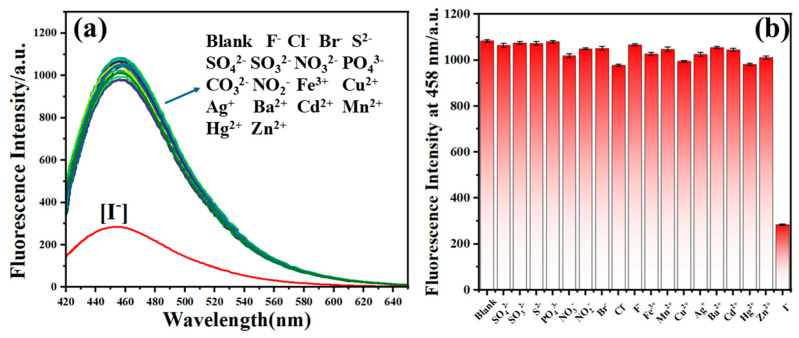
(**a**) The fluorescence responses of the AgFeS_2_ QDs (2 mL) dispersed in ethanol:water = 1:4 towards different interfering substances (50 μM). Excitation wavelength: 368 nm. (**b**) A column graph of the emission changes in the presence of interfering substances (50 μM).

In our study, a facile, effective and low-cost approach has been established and the detection of iodine in an aqueous solution has been explored. As can be seen from the PL spectra, the emission peak intensities of the AgFeS_2_ QDs decreased with increased I- ion concentration. When studying the selectivity of the AgFeS_2_ QDs for I^−^, the nanomaterial showed negligible fluorescence changes in the presence of a group of ions (SO_4_^2−^, SO_3_^2−^, S^2−^, PO_4_^3−^, NO^3−^, NO^2−^, F^−^, Cl^−^, Br^−^, Fe^3+^, Mn^2+^, Cu^2+^, Ag^+^, Ba^2+^, Cd^2+^, Zn^2+^ and Hg^2+^), as shown in Figure 6, which suggests that the AgFeS_2_ QDs aqueous solution possesses excellent selectivity toward I-. Moreover, interference experiments to identify iodide have been performed. As shown in Figure S6, the emission intensity exhibited no specific changes in the presence of other interfering substances. But the fluorescence was sharply quenched after the incorporation of iodide. The experimental results confirm that the nanoprobes can be expected to provide an important platform for the detection of iodide ions in complex media.

**Figure 7:**
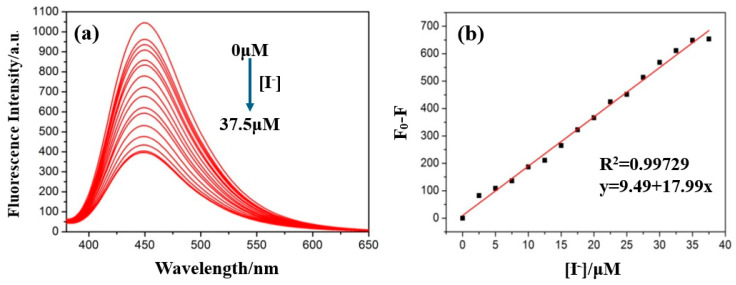
(**a**) The fluorescence responses of the AgFeS_2_ QDs (2 mL) dispersed in ethanol:water = 1:4 towards iodide from 0 to 37.5 μM (Ex = 368 nm). (**b**) The emission band at 458 nm (F_0_-F) versus the iodide concentration in ethanol:water = 1:4 (F and F_0_ are the fluorescence intensity of the AgFeS_2_ QDs in the presence and absence of I^−^).

With the rapid development of accurate determination, the performance of nanodomains in the recognition of guest species has been recorded. Based on the above fluorescence evolution changes, various concentrations of iodide ion solution were added to the aqueous solution of the probe. As shown in Figure 7a, the fluorescence signal of the probe gradually decreased as the concentration of I- varied from 0 to 37.5 μM. A good linear correlation in the range of 0 to 37.5 μM with a correlation coefficient square (R^2^) of 0.99792 was obtained, as shown in Figure 7b, while the detection limit for I- using the probe was calculated to be 0.99 μM based on 3σ/k, indicating that the functional quantum dots could be potentially used in the measurement of I^−^. With the aim of evaluating their accuracy, a variety of probes including MOFs, COFs and alloys have been recorded and compared. Although the detection limit for the AgFeS_2_ quantum dots was not the most sensitive one (Table S1), it still gave a highly acceptable value, demonstrating that the sensor possesses a strong affinity for iodide. The response time for the sensitive detection of iodide was 30 s.

**Figure 8:**
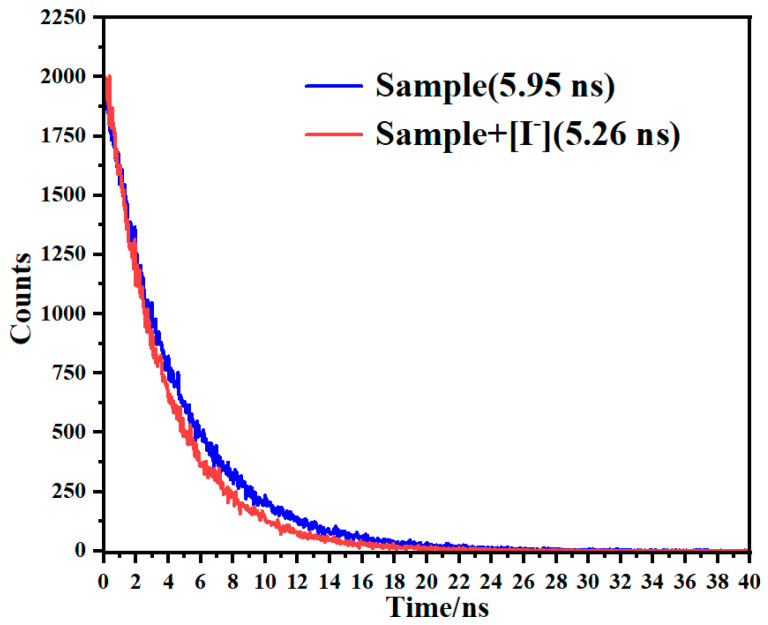
The evolution of the PL decay dynamics of the AgFeS_2_ QDs samples in the absence (blue) and the presence (red) of 50 μΜ I^−^ ions.

In regard to mechanism studies, the fluorescence of a probe can be generally judged by either dynamic quenching or static quenching processes. To explore the switch behavior of the AgFeS_2_ QDs in the aqueous solution, the fluorescence lifetime (τ) of the QDs before and after the addition of iodide ions in the aqueous solution was carefully studied [38,39]. As shown in Figure 8, based on a monoexponential fit, the fluorescence lifetime (τ) of the QDs was 5.95 ns and 5.26 ns in the absence or presence of iodide ions; the corresponding values, although possessing slight changes, were almost essentially the same order of magnitude. The invariable lifetime showed a static quenching process between the AgFeS_2_ QDs and the iodide ions. This fact suggests that no dynamic change process could be involved during the determination of guest ions.

**Figure 9:**
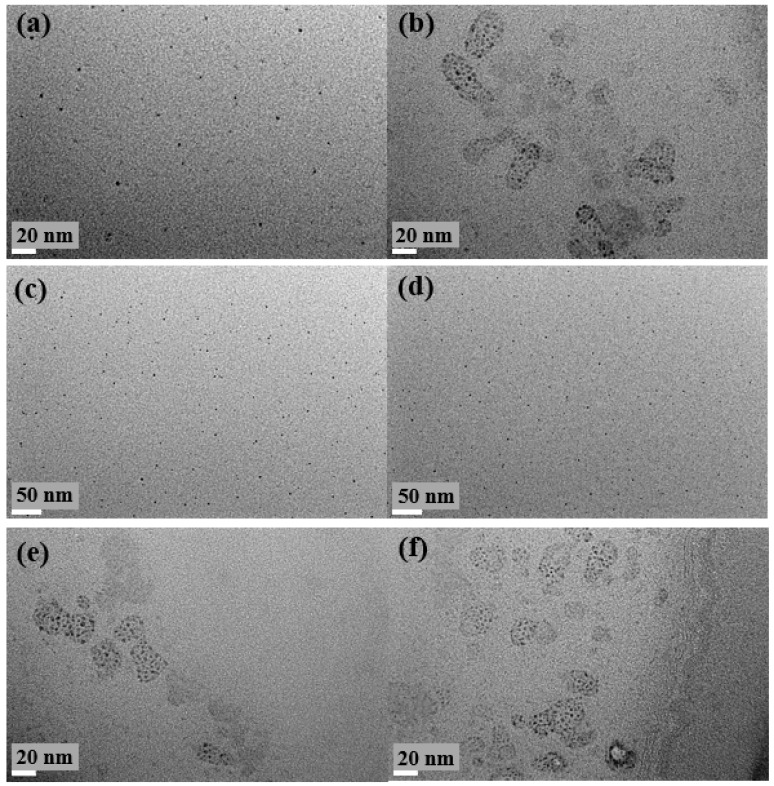
TEM images of (**a**) the AgFeS_2_ QDs dispersed in ethanol:water = 1:4 and (**b**) the AgFeS_2_ QDs with the addition of 50 μΜ I^−^ ions. (**c**) AgFeS_2_ QDs dispersed in ethanol:water = 1:4 and maintained at room temperature for 10 h; (**d**) AgFeS_2_ QDs dispersed in ethanol:water = 1:4 and maintained at room temperature for 24 h; (**e**) AgFeS_2_ QDs with the addition of 50 μΜ I^−^ ions for 10 minutes; (**f**) AgFeS_2_ QDs with the addition of 50 μΜ I^−^ ions for 1 h.

To demonstrate the underlying mechanism of the static quenching process with the addition of iodide ions, we used transmission electron microscopy (TEM) to describe the changes in the surface size of quantum dots. As shown in Figure 9, in the absence and presence of 50 μM iodide ions, the morphology and shape were drastically changed. The results support the idea that AgFeS_2_ QDs and I^−^ hybrid systems can be used as potential platforms for identifying iodide ions. Iodide-induced fluorescence quenching is based on the high affinity between I^−^ and Ag^+^ ions; the low solubility product constant (K_sp_) of AgI leads to the structure change. Therefore, the binding interaction between I^−^ ions and Ag^+^ ions causes aggregation among the two species, resulting in an “on-off” signal change. The microstructure analysis demonstrated that the tiny particles could turn from a mono-dispersed form to one with large clusters and the aggregation causing quenching would be obtained (Figure S7).

**Figure 10:**
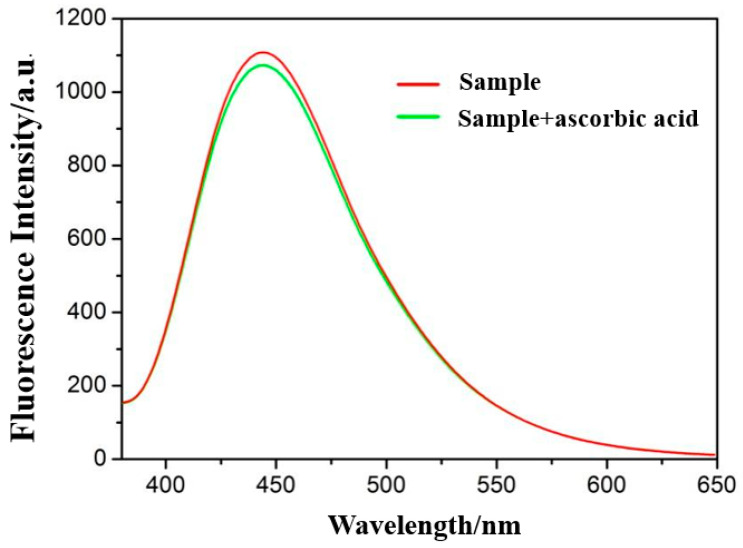
The effect of ascorbic acid (50 μM) on the fluorescence emission spectra of the AgFeS_2_ QDs dispersed in ethanol:water = 1:4.

In order to show the practical uses of the fluorescent sensor with real samples, we have used the nanoprobe to detect I^−^ ions in the form of a salt sample. In many countries, potassium iodate has been incorporated into salt samples as a source of iodine, because of its high chemical stability. For this reason, we selected this method to measure iodine after converting iodate to iodide, using ascorbic acid as the reductant [40]. To rule out the effect of ascorbic acid on the sample, the fluorescence of the AgFeS_2_ QDs was measured in the absence and presence of ascorbic acid, as shown in Figure 10, suggesting that the type of reductant reagent has negligible influence on the photophysical properties of the AgFeS_2_ QDs. Accordingly, the concentration of I^−^ in the samples was analyzed by the method of standard addition (Table 1). The above results suggest that this fluorescent probe could be used for the accurate measurement of iodide in salt samples.

## 4. Conclusions

In summary, a synthetic pathway was developed to obtain AgFeS_2_ nanoparticles at a low temperature, employing oleylamine as the precursor. Each element of the AgFeS_2_ nanoparticles has been confirmed using PhenomProX SEM measurements. The magnetic properties of the AgFeS_2_ nanoparticles were shown by employing a vibrating sample magnetometer and the AgFeS_2_ NCs were shown to be ferromagnetic at low temperatures. The AgFeS_2_ QDs can present a bright blue emission in aqueous solutions, which is effectively quenched upon the addition of I^−^. The detection limit of I^−^ was detected to be 0.998 μM with a good linear correlation. Furthermore, the probe could be applied for detecting I^−^ in real salt samples. It is expected that this strategy may offer an alternative approach for achieving low-cost, highly sensitive and selective sensors in environmental and biological fields.

## Figures and Tables

**Table 1:** The content of iodide in iodine-fortified salt samples was measured by using a fluorescent probe of AgFeS_2_ QDs.

Samples	Added (μM)	Found (μM)	Recovery [%]	RSD (n = 3, %)
1	5	5.02	100.4%	2.52%
2	15	15.21	101.4%	1.36%
3	20	19.87	99.35%	2.98%

## Data Availability

The data will be available upon request to the corresponding author.

## Supplementary Materials

The following supporting information can be downloaded at: https://www.mdpi.com/article/10.3390/molecules29194751/s1, Figure S1: (a) Elemental distribution images of the sample; (b–d) Areal density of each of the elements extracted from the electron energy loss spectroscopy spectrum image. Figure S2: XPS analysis of (a) full survey, (b) silver 3d, (c) iron 2p and (d) sulfur 2p. Figure S3: Zeta potentials of AgFeS_2_ Nanoparticles in ethanol. Figure S4: Fluorescence emission spectra of AgFeS_2_ QDs samples with different excitation spectra from 300 nm to 420 nm (emission center: basically concentrated in 450 nm). Figure S5: Fluorescence intensity of the AgFeS_2_ QDs with different pH values at 368 nm of the fluorescence excitation spectra. Figure S6: Interference experiments of fluorescence recognition of I− (25 µM) and other species (50 µM) (1. Blank, 2. F^−^, 3. Cl^−^, 4. Br^−^, 5. S^2−^, 6. SO4^2−^, 7. SO3^2−^, 8. NO3^2−^, 9. PO4^3−^, 10. CO3^2−^, 11. NO^2−^, 12. Fe^3+^, 13. Cu^2+^, 14. Ag^+^, 15. Ba^2+^, 16. Cd^2+^, 17. Hg^2+^, 18. Zn^2+^). Figure S7: Schematic illustration of the fluorescence change of AgFeS_2_ QDs after addition of iodide. Table S1: Comparison between the current method and the reported literature for the detection of iodide.
