# Supplementary material for: Fast and Sensitive Determination of Iodide Based on Ternary Chalcogenides Nanoparticles
Source: Molecules. 2024 Oct 8;29(19):4751. doi: 10.3390/molecules29194751 (PMC11478270; doi:10.3390/molecules29194751)
Supplement: Supplementary file 1 [file molecules-29-04751-s001.zip › molecules-3239301-supplementary.pdf]

Supporting information for

Fast and Sensitive Determination of Iodide Based on Ternary Chalcogenides Nanoparticles

Zhitai Wang [1], Nengtao Wu [2], Weihao Wang [2], Yaozheng Hu [1], Zhijie Luo [2], Yuhui Zheng [2,*] and Qianming Wang [2,*]

[1] School of Materials Science and Engineering, Nanchang Hangkong University, Nanchang 330063, China; wangzt@nchu.edu.cn (Z.W.)

[2] School of Chemistry, Guangzhou Key Laboratory of Analytical Chemistry for Biomedicine, South China Normal University, Guangzhou 510006, China

* Correspondence: yhzheng78@scnu.edu.cn (Y.Z.); qmwang@scnu.edu.cn (Q.W.)

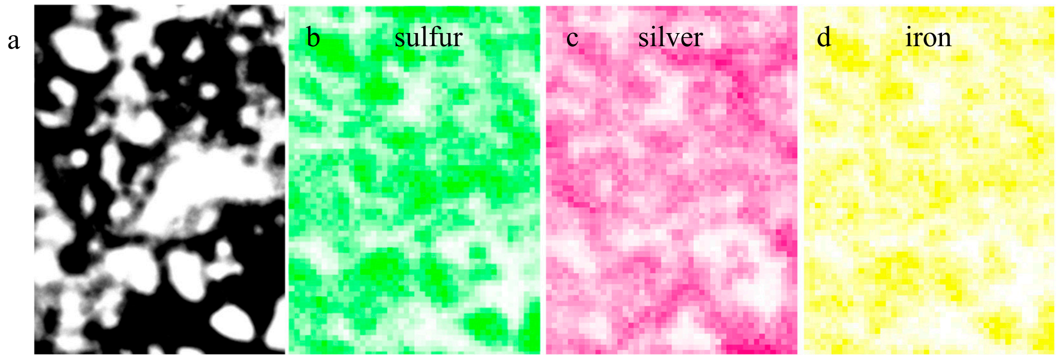

Figure S1 (a) Elemental distribution images of the sample; (b-d) Areal density of each of the elements extracted from the electron energy loss spectroscopy spectrum image.

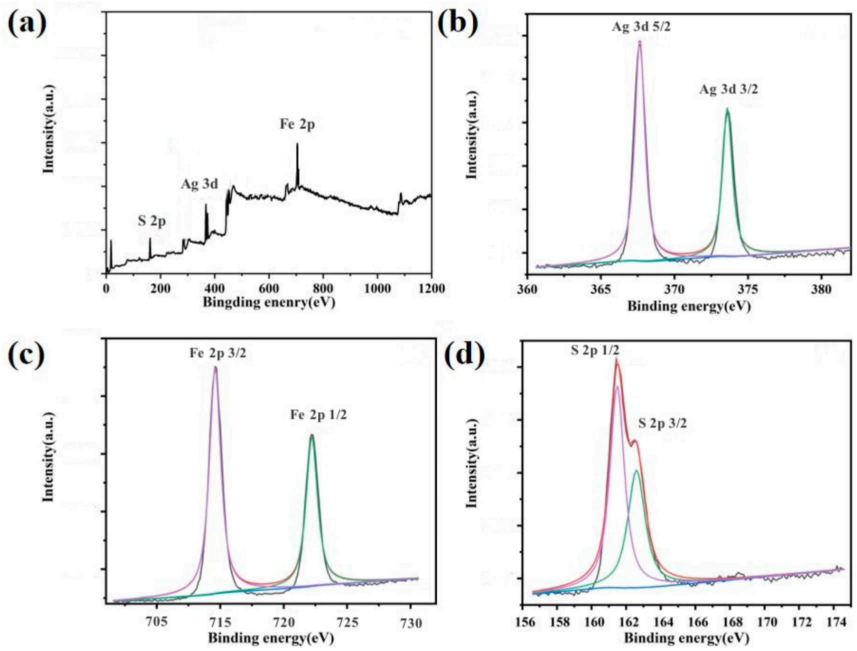

Figure S2 XPS analysis of (a) full survey, (b) silver 3d, (c) iron 2p and (d) sulfur 2p

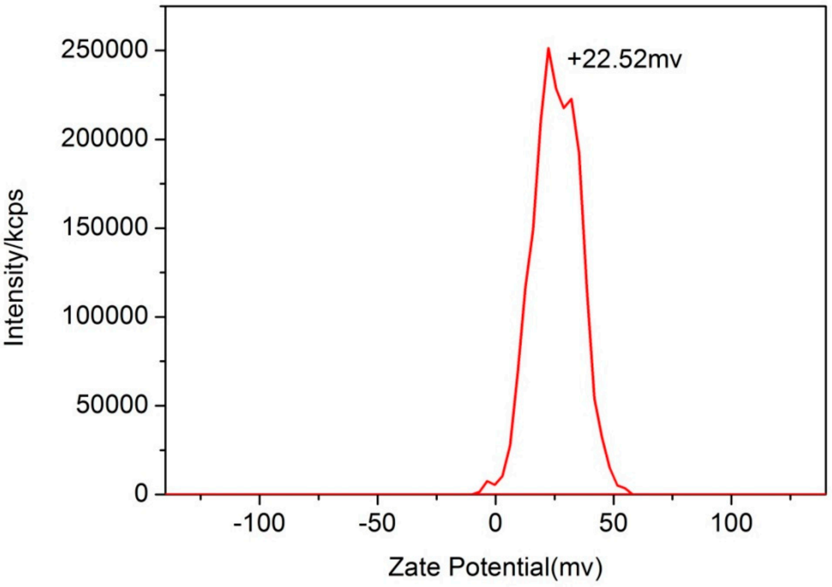

Figure S3 Zeta potentials of $AgFeS_2$ Nanoparticles in ethanol.

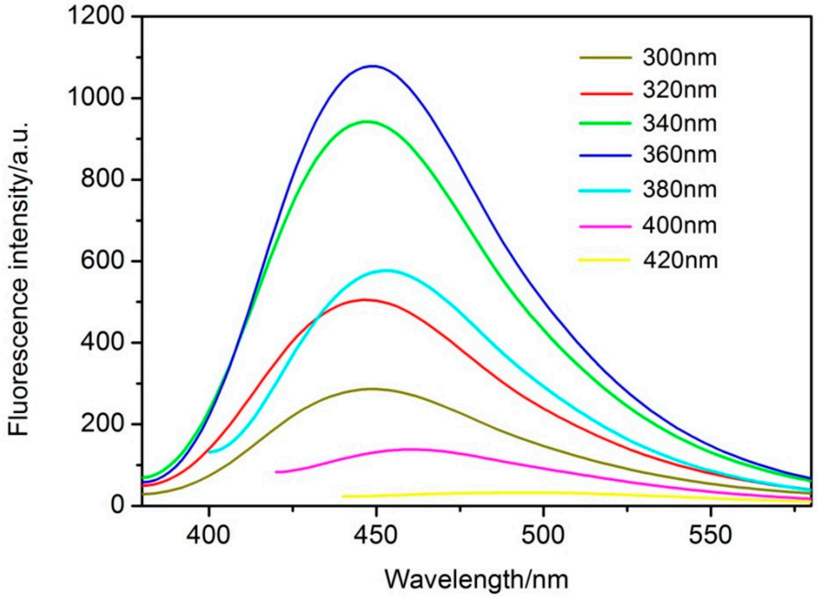

Figure S4 Fluorescence emission spectra of $AgFeS_2$ QDs samples with different excitation spectra from 300 nm to 420 nm (emission center: basically concentrated in 450 nm).

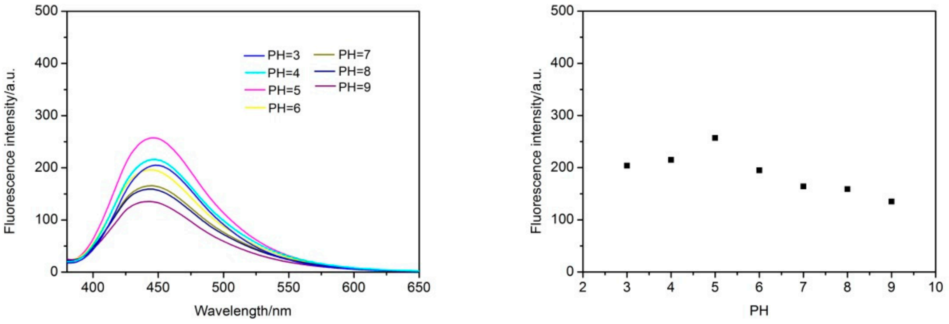

Figure S5 Fluorescence intensity of the $AgFeS_2$ QDs with different pH values at 368 nm of the fluorescence excitation spectra.

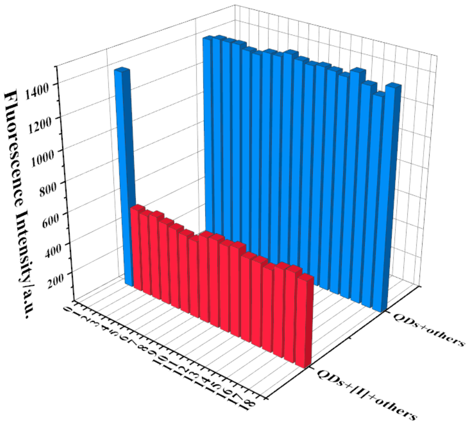

Figure S6 Interference experiments of fluorescence recognition of I^- (25 μM) and other species (50 μM) (1. Blank, 2. F^-, 3. Cl^-, 4. Br^-, 5. S^{2-}, 6. SO_4^{2-}, 7. SO_3^{2-}, 8. NO_3^{2-}, 9. PO_4^{3-}, 10. CO_3^{2-}, 11. NO_2^-, 12. Fe^{3+}, 13. Cu^{2+}, 14. Ag^+, 15. Ba^{2+}, 16. Cd^{2+}, 17. Hg^{2+}, 18. Zn^{2+})

Table S1 Comparison between the current method and the reported literature for the detection of iodide

Materials	Detection Limit	References
Au-Pt alloy	0.29 µM	S1
poly(diallyldimethylammonium chloride)	1.31 µM	S2
azido pyrazole-chalcone derivatives	2.5 mM	S3
TT-COF(Zn)	3.15 µM	S4
Lanthanide MOFs	4.7 µM	S5
Ag complex	5.31 µM	S6
Au-CN	8.9 µM	S7
AgFeS2 quantum dots	0.99 µM	This work

[S1] Li, H.; Zhang, Y.; Zhang, J.; Huo, K.; Gu, J.; Zhou, Y.; Liu, Y.; Liu, Y.; Liu, X., Bimetallic-based colorimetric sensor for highly selective, stable and sensitive detection of iodide ions. *Microchemical Journal* **2024,** 199.

[S2] Fukushima, Y.; Aikawa, S., Colorimetric detection of iodide ion by a nuclear fast red-based Hg2+ complex in aqueous media. *Tetrahedron Letters* **2021,** 67.

[S3] Joshi, R. J.; Varu, H. L.; Bhalodia, J. J.; Ambasana, M. A.; Bapodra, A. H.; Kapuriya, N. P., Highly selective fluorescence sensor based on azido pyrazole-chalcone conjugates for rapid detection of iodide ion. *Results in Chemistry* **2024,** 7.

[S4] Wei, M.-J.; Wei, Z.-Q.; Li, J.; Yu, L.; Zhang, S.-F.; Cheng, F.; Li, H.-Y.; Kong, F.-Y.; Wang, W., Covalent organic framework with extraordinary intrinsic catalytic activity for electrochemical sensing of iodide ions. *Microchemical Journal* **2024,** 200.

[S5] Zhong, X.; Li, C.; Chen, H.; Deng, P., Lanthanide doped metal−organic framework: novel turn-on fluorescent sensing of iodide in kelp and seawater samples. *Microchemical Journal* **2024,** 202.

[S6] Goh, H.; Nam, T. K.; Singh, A.; Singh, N.; Jang, D. O., Dipodal colorimetric sensor for Ag+ and its resultant complex for iodide sensing using a cation displacement approach in water. *Tetrahedron Letters* **2017,** 58, (11), 1040-1045.

[S7] Saha, C.; Ghosh, S. K.; Kumari, P.; Perla, V. K.; Singh, H.; Mallick, K., Electrocatalytic efficiency of carbon nitride supported gold nanoparticle based sensor for iodide and cysteine detection. *Anal Biochem* **2024,** 696, 115660.

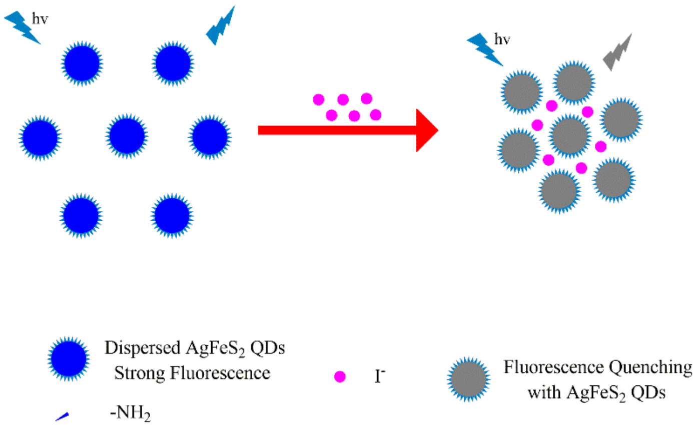

Figure S7 Schematic illustration of the fluorescence change of $AgFeS_2$ QDs after addition of iodide.
